# Editorial: Psychological Dimensions in Human Sexual Health and Behavior

**DOI:** 10.3389/fpsyg.2021.739708

**Published:** 2021-08-25

**Authors:** Filippo Maria Nimbi, Peer Briken, Carmita H. N. Abdo, Joana Carvalho

**Affiliations:** ^1^Department of Dynamic, Clinical and Health Psychology, Sapienza University of Rome, Rome, Italy; ^2^Institute for Sex Research, Sexual Medicine, and Forensic Psychiatry, University Medical Centre Hamburg-Eppendorf, Hamburg, Germany; ^3^Department of Psychiatry, Faculty of Medicine, University of São Paulo, São Paulo, Brazil; ^4^CPUP: Center for Psychology of Porto University, Faculty of Psychology and Educational Sciences, Porto University, Porto, Portugal

**Keywords:** sexuality, psychological, psychology, sexual health, clinical psychosexology

Human sexuality is still an underexplored world, subjected to great taboos and controversies over the decades, representing one of the most challenging areas of research and facing countless political and social demands. In this sense, leading organizations such as the World Health Organization (WHO, 2014) and the World Association for Sexual Health (WAS, 2014) have established sexual health and well-being as human rights and key goals to be achieved worldwide to improve the health-related quality of life. The new message that is being carried out by these entities, is that sexual health not only concerns reproductive issues, sexual problems, sexual violence, and sexually transmitted infections, but also positive aspects related to intimate relationships, pleasure, consenting sexual relationships, gender and sexual orientation variety, and sexual functioning among other aspects.

The current special issue on “Psychological Dimensions in Human Sexual Health and Behavior” represents an important step toward a broader biopsychosocial understanding of human sexuality (Berry and Berry, 2013). After a major focus on organic factors underpinning sexual difficulties and behavior, researchers have recognized the need of targeting the psychological factors, and the interplaying role between organic, psychological, and social aspects affecting sexual health and well-being (Assalian, 2013; Brotto et al., 2016).

The psychological dimensions are being considered as central elements in the international guidelines for clinical intervention in sexual difficulties and dysfunctions, sexual health promotion programs, and sexual education (DeRogatis, 2008; Althof et al., 2012; Bitzer et al., 2013; Fugl-Meyer et al., 2013; Laan et al., 2013; McMahon et al., 2013; Mulhall et al., 2013; WHO, 2018). These psychological dimensions have been framed within conceptualizing models of human sexual response, often including cognitions, emotions, personality traits, psychopathology, socio-cultural, and relational variables influencing sexual functioning and behavior.

In this Research Topic, readers will find interesting and innovative contributes to the understanding of the role of some psychological components in peculiar aspects of sexuality such as cognitive processing and response to sexual stimuli, sexual satisfaction, and adherence to Sexual Double Standards (SDS), coming out in LGBTQI+ population, polyamory, sexual violence, and trauma.

Understanding the processing of sexual stimuli has become prominent in human sexuality research, since it may explain the arousal process and the cognitive mechanisms underlying the sexual response (Huberman, 2021). Erotic processing seems to be very relevant in our brain. Novák et al. reported the absence/inconsistency of spatial attention bias to sexual images and suggested that sexual stimuli are prioritized in memory and cognitive processes compared to other stimuli. Moreover, literature has shown that cultural factors play a primary role in sexual stimuli processing (Rupp and Wallen, 2008). Recognizing the need of including cultural relevance in imaging studies, Cui et al. validated and shared a sexual stimuli database, useful for further research in eastern Asian cultural settings, showing some interesting gender differences in sexual arousal, pleasantness, and sexual attractiveness ratings.

How adherence to gender roles may affect psychology in sexuality represent one of the main challenges in sex research. Álvarez-Muelas et al. showed that relationship satisfaction may be the main predictor of sexual satisfaction and vary according to gender and SDS adherence. These results suggest the urgency to investigate how people internalize attitudes toward the SDS in future studies.

The role of attitudes is also relevant when it comes to LGBTQI+ and Polyamory. For example, Rosati et al. extended the current knowledge on the role of coming out and minority stress experiences among different generations of LGBQ+ people. On average, older adults became self-aware and disclose at a later age than younger men, they seem to be more Catholic and came out more frequently to their Catholic community, with reactions ranging from total acceptance to open rejection. Focusing on women, Baiocco et al. reported that lesbian women had their coming out to both their parents more often than bisexual ones, reporting lower levels of internalized sexual stigma and more positive attitudes toward lesbian/bisexual identity. These new studies contribute to our understanding of coming out peculiarities in varied forms of sexual and relational expressions. Moors et al. with their focus on polyamory, showed that a significant percentage of Americans desire to engage and/or has already engaged in polyamory. Few sociodemographic variables were related to this interest and given that relational intimacy is an important part of most people's lives, understanding the varied ways in which people cross in their intimacy is crucial for social, psychological, and sexological fields (Vaughan et al., 2019).

A significant focus in this special issue has been given to sexual offenders and victims. This is still a relevant topic under different perspectives. International reports (UNICEF, 2017; WHO, 2021) draw an alarming figure with respect to the rate of abuse in minors and adults, on the need to find more effective prevention strategies and programs to support victims, as well as the management and treatment of offenders. In this context, Barroso et al. focus on specific kind of abuse such as the study on sexting in adolescence in terms of emotional and behavioral problems, potential markers of psychopathy, childhood trauma and maltreatment, and different forms of aggression. The criminal responsibility of sexual offenders with paraphilic disorders is also a current challenge. Dobbrunz et al. presented an assessment following a two-stage method for the severity of a paraphilic disorder and the criteria for/against diminished capacity according to the German legal system, giving ground to an empirically based assessment of criminal responsibility.

From the victims' side, the psychological factors that may improve/worsen the outcome of traumatic conditions such as child sexual abuse are of extreme interest to offer tailored support weighting to the individual resources/weaknesses (Daigneault et al., 2007). Ensink et al. stress the attention on the possible interaction between sexual abuse history and attachment security, showing that sexually abused children with insecure attachment seem to be more at risk for post-traumatic symptoms. Also, in later stages of life, sexual traumas can have profound consequences on quality of life and sexual relationships. Almås and Pirelli Benestad give specific directions for psychological treatments integrating different clinical approaches, highlighting how traumatized people need particular attention to safety, respect, and acceptance.

The journey to understand sexuality is still long and tortuous, but this special issue represents a small significant step in this direction. Enjoy the reading.

## Author Contributions

FMN wrote the first draft of the manuscript. All authors have reviewed, discussed and accept the final version.

## Conflict of Interest

The authors declare that the research was conducted in the absence of any commercial or financial relationships that could be construed as a potential conflict of interest.

## Publisher's Note

All claims expressed in this article are solely those of the authors and do not necessarily represent those of their affiliated organizations, or those of the publisher, the editors and the reviewers. Any product that may be evaluated in this article, or claim that may be made by its manufacturer, is not guaranteed or endorsed by the publisher.
